# Exploring alexithymia with the French Perth alexithymia questionnaire: latent structure, profiles, and links with affective outcomes

**DOI:** 10.3389/fpsyg.2025.1615612

**Published:** 2025-06-17

**Authors:** Carole Fantini-Hauwel, Cassandra Gois, Olivier Luminet, Emilie Banse, Alix Bigot, Xavier Noel, David A. Preece

**Affiliations:** ^1^Research Center of experiMEntAl, CogNItive and CliNical PsycholoGy (MEANING), Université Libre de Bruxelles, Brussels, Belgium; ^2^Research Institute for Psychological Sciences, UCLouvain, Louvain la Neuve, Belgium; ^3^Fund for Scientific Research (FRS-FNRS), Brussels, Belgium; ^4^Laboratoire de Psychologie Médicale et d'Addictologie, ULB Neuroscience Institute, CHU Brugmann, Université Libre de Bruxelles, Brussels, Belgium; ^5^School of Population Health, Curtin University, Perth, WA, Australia; ^6^Department of Psychology, Stanford University, Stanford, CA, United States; ^7^School of Psychological Science, The University of Western Australia, Perth, WA, Australia

**Keywords:** alexithymia, questionnaire, measurement, attention-appraisal model, emotional awareness, emotion, psychometric, latent profile

## Abstract

**Introduction:**

Alexithymia is of high clinical interest and its measurement remains an important evolving area of research. The Perth Alexithymia Questionnaire (PAQ) is a self-report measure designed to enable facet-level and valence-specific assessments of alexithymia. Here we aimed to introduce a French version of the PAQ, examine its psychometric performance, and use the PAQ to further explore the nature of the alexithymia construct.

**Method:**

Participants in Belgium (*N* = 481) completed the PAQ and other self-report measures. Factor structure, reliability, and concurrent/discriminant validity were assessed, as well as an exploration of alexithymia profiles with latent profile analysis (LPA).

**Results:**

Confirmatory factor analysis confirmed the PAQ had a theoretically congruent factor structure. All PAQ scores had high reliability and showed good concurrent validity with other measures of alexithymia, emotion regulation, and psychopathology. Sound discriminant validity was also established. Our LPA extracted eight profiles, highlighting the value of facet-level and valence-specific analysis of alexithymia. Some profiles had difficulties in all facets of alexithymia and both valence domains, whereas others had difficulties only in the appraisal of negative emotions.

**Conclusion:**

Overall, our results indicate that the French PAQ has strong psychometric properties, and that facet-level and valence-specific assessments can be valuable in further understanding the alexithymia construct.

## 1 Introduction

Alexithymia, meaning “no words for emotions” in Greek Sifneos (1973), is a trait characterized by a set of at least three emotion processing deficits: difficulties identifying one's own feelings (DIF); difficulty describing one's own feelings (DDF); and an externally oriented thinking style (EOT), whereby one rarely focuses attention on their internal emotional states (Preece et al., 2017). Some authors also consider constricted imaginal processes (rare daydreaming) part of the alexithymia construct (e.g., Taylor and Bagby, 2021), though there remains debate in the field on this point (for a review, see Luminet and Nielson, 2024). First coined in the 1970's based on observations of psychiatric patients (Sifneos, 1973), alexithymia has since been established as an important risk factor for a range of psychopathologies, including depression, anxiety disorders, substance use, eating disorders, psychosomatic disorders, and personality disorders (Speranza et al., 2005; Chaim et al., 2024; Panayiotou, 2018). Alexithymia is normally distributed in the general population, with around 10% of people having problematically high levels (Luminet et al., 2021a). Sometimes research compares “alexithymic” and “non-alexithymic” groups, however, the categorical approach is not the most appropriate as alexithymia is better understood as a stable individual difference, present at some degree in all people with different variation among the three facets (Luminet and Nielson, 2024). This explains why two similar alexithymia total scores do not always reflect exactly the same pattern of alexithymia at the facet level (Keefer et al., 2019).

Among existing alexithymia theoretical frameworks (Taylor and Bagby, 2021; Luminet et al., 2021b), one model that can be useful for understanding alexithymia is the attention-appraisal model of alexithymia (Preece et al., 2017; Preece and Gross, 2023), which maps the construct within contemporary affective science frameworks (e.g., the process model of emotion regulation; Gross, 2015). This model outlines how emotions are processed via four-stage situation-attention-appraisal-response sequences. When an emotion (situation stage) is present, to process it, one must first focus attention on the emotion (attention stage), appraise it in terms of what it is and what it means (appraisal stage), and based on that appraisal, one then may activate a goal to try to regulate that emotion (response stage, i.e., emotion regulation). Within this framework, alexithymia can be understood as one's degree of difficulties at the attention (i.e., EOT) and appraisal (i.e., DIF, DDF) stages of emotion processing. Empirical data support that difficulties at these stages are caused by a combination of ability deficits (i.e., low theoretical knowledge of emotions, underdeveloped cognitive structures for processing emotions; Lane and Schwartz, 1987) and/or avoidance (i.e., to cope with emotions, avoiding focusing on them; Panayiotou et al., 2015). Research indicates that individuals with high levels of alexithymia exhibit deficits in early attentional processing stages, characterized by the allocation of fewer attentional resources to emotional stimuli (Lee and Lee, 2024), particularly in relation to EOT (Wiebe et al., 2017). Furthermore, alexithymia has been linked to biases in the appraisal of emotional experiences, including dimensions of valence, frequency, and intensity. Specifically, individuals with high alexithymia scores tend to overestimate negative stimuli and underestimate positive stimuli (Luminet et al., 2004; Rigby et al., 2020; Koven, 2014). However, these effects may depend on contextual factors and the specific facets of alexithymia. For a comprehensive review of studies examining the relationship between alexithymia, emotional appraisals, and attention, among other related concepts, refer to the review by Luminet et al. (2021b) suggesting that alexithymia implies a deficit or an over-responding pattern in emotional processing, depending on contextual circumstances. Because accurate attention to and appraisal of emotions is an important factor behind emotion regulation decisions, alexithymia appears to impair down-stream emotion regulation processes, in turn predisposing people to psychopathologies characterized by emotion dysregulation (for a review, see Preece and Sikka, 2024). Indeed, those with high alexithymia usually report experiencing more frequent and intense negative affect and less positive affect (Fantini-Hauwel et al., 2015).

### 1.1 Assessing alexithymia

The assessment of alexithymia is therefore important in research and clinical settings. This is usually done via self-report questionnaires. One of the earliest and most popular alexithymia questionnaires has been the 20-item Toronto Alexithymia Scale (TAS-20), introduced in 1994 (Bagby et al., 1994). While the TAS-20 continues to make important contributions to the field, it was originally designed to provide only a total score as an overall marker of alexithymia. The TAS-20 developers have recommended against extracting any subscale scores for the specific facets of alexithymia (Bagby et al., 2007; Carnovale et al., 2021). If facet scores are extracted, the EOT dimension has low reliability (α < 0.70; Kooiman et al., 2002) below standard psychometric cut-offs for use of a score in research or clinical settings (Groth-Marnat, 2009). However, there is evidence from factor analytic studies indicating that a structure with the three facet dimensions alone, or nested within a general factor, can demonstrate a good model fit, emphasizing that consideration of the facet dimensions may help to provide a fuller picture of alexithymia than a general score considered alone (Schroeders et al., 2022; Müller et al., 2003). There is also evidence that introducing a method factor (loading on all revers-scored items) improves the fit of TAS-20 factor models (e.g., Gignac et al., 2007; Watters et al., 2016), suggesting that the high proportion of reverse-scored items in the EOT facet may partly explain the lower reliability observed in this dimension. Lastly, there is factor analytic evidence that the DIF items may be confounded by distress, i.e., measure people's current distress levels rather than just alexithymia (Preece et al., 2024b; Leising et al., 2009); as some items ask directly about symptoms that tap with anxiety (e.g., “I have physical sensations that even doctors don't understand”; see also Veirman et al., 2021).

Given the multidimensional nature of the alexithymia construct, recent trends in the field have increasingly emphasized the examination of alexithymia at the facet level. This approach highlights the distinct contributions of individual facets to emotional and cognitive processes, which hold particular significance for both research and clinical applications (for a review, see Luminet and Nielson, 2024). Hence, there is a need for more tools designed to enable robust facet (subscale) level analysis. One such tool designed for this purpose is the more recently introduced Perth Alexithymia Questionnaire (PAQ). This paper aims to introduce the first French version of the PAQ, evaluate its psychometric properties, and in so doing, use it to further explore the nature of the alexithymia construct.

### 1.2 The Perth alexithymia questionnaire

The PAQ (Preece et al., 2018) is a 24-item self-report measure of alexithymia based on the attention-appraisal model of alexithymia. It aims to assess the DIF, DDF, and EOT facets of alexithymia. One novel aspect of the PAQ is that, for the appraisal dimensions, it has valence-specific subscales, specific to either negative or positive emotions. Conceptually, this aligns with findings from the broader emotion field that emotional constructs can often function differently across negative and positive emotions (e.g., Gross, 2015; Becerra et al., 2020), as well as neuroanatomical data suggesting that alexithymia often involves deficits processing emotions in both valence domains (Van der Velde et al., 2013). Traditionally, most existing emotion processing studies have focused mainly on negative affect, while there is growing evidence that difficulties regulating positive emotions are associated with worse outcomes such as gambling, risky behaviors (Cyders and Smith, 2008; Cyders et al., 2007), increased sympathetic nervous system activation (Gross and Levenson, 1997), bipolar disorders (Velotti et al., 2020), and decreased wellbeing (Yu et al., 2023). Thus, there may be value in considering the positive valence domain in alexithymia assessments too.

Consequently, the PAQ has 5 subscales: *Negative-Difficulty Identifying Feelings* (N-DIF; “When I'm feeling bad, I can't make sense of those feelings”), *Positive-Difficulty Identifying Feelings* (P-DIF; “When I'm feeling good, I can't tell whether I'm happy, excited, or amused”), *Negative-Difficulty Describing Feelings* (N-DDF; “When I'm feeling bad, I can't talk about those feelings in much depth or detail”), *Positive-Difficulty Describing Feelings* (P-DDF; “When something good happens, it's hard for me to put into words how I'm feeling”), and *General-Externally Oriented Thinking* (G-EOT; “I don't pay attention to my emotions”). These subscales can also be combined into a range of theoretically meaningful composite scores, such as N-DIF and N-DDF scores being combined into a *Negative-Difficulty Appraising Feelings* (N-DAF) composite, and P-DIF and P-DDF scores being composite into a *Positive-Difficulty Appraising Feelings* (P-DAF) composite, reflecting their common conceptual mapping to the appraisal stage of emotion processing (Preece and Gross, 2023). All items can also be summed into a total score as an overall marker of alexithymia.

First developed in English, the PAQ has since been translated, validated and used in a range of different language versions and cultures, for example, including samples from Australia (Greene et al., 2020), the United States (Preece et al., 2020), Singapore (Chan et al., 2023), China (Cai et al., 2024), Chile (Becerra et al., 2021), Spain (Kiskimska and Martínez-Sánchez, 2023), and Poland (Larionow et al., 2022). Factor analyses of the PAQ have consistently supported a 5-factor solution, comprised of 5 correlated factors corresponding to the intended subscales. This model tends to fit better than simpler models that do not distinguish between DIF, DDF, and EOT, or models which do not distinguish between the negative and positive valence domains. The DIF and DDF factors within each valence domain tend to be highly correlated, and hence models that combine them (i.e., into N-DAF and P-DAF scores that capture the appraisal stage of emotion processing more broadly) are also often tenable, but separating DIF and DDF has nonetheless usually added statistical value to the factor solution. These patterns have been supported across a range of community, student, and clinical samples (e.g., Fynn et al., 2022; Trimble et al., 2024), with evidence also for the factor structure being invariant across people of different cultures, genders, ages, and education levels (Chan et al., 2023; Mazidi et al., 2023). Studies that have tested a bifactor model, where a “general alexithymia” factor is represented alongside the narrow subscale factors, have also found support for this presence of a general factor (Preece et al., 2018; Cai et al., 2024; Becerra et al., 2021). Thus, existing work with the PAQ supports that alexithymia is a coherent multidimensional construct.

Examinations of reliability have consistently found that all PAQ subscales and composites have high levels of internal consistency (Greene et al., 2020; Mazidi et al., 2023; Ferguson et al., 2023), with evidence for test-retest stability over time (Asl et al., 2020; Larionow et al., 2023). PAQ scores also appear to correlate in expected directions with a range of other constructs or external markers. The PAQ total score correlates highly (around 0.75–0.80) with the total score of other alexithymia measures (Preece et al., 2020; Chan et al., 2023). PAQ scores are also associated with more overall emotion regulation difficulties, and higher use of avoidant and maladaptive emotion regulation strategies (e.g., Mehta et al., 2024). In line with the status of alexithymia as a transdiagnostic risk factor for psychopathology, PAQ scores are consistently associated with higher levels of symptoms of a range of disorders, including depression, anxiety, personality disorders, somatoform disorder, eating disorders, and substance use (Greene et al., 2020; Cai et al., 2024). PAQ facet level scores have been found to contribute unique variance to the prediction of psychopathology, including prominent variance accounted for by difficulties in the positive valence domain (Preece et al., 2024a; Winterstein et al., 2024). Importantly, all examinations of the PAQ's discriminant validity have been supportive to date, with the PAQ appearing to measure an alexithymia construct that is separable from people's current levels of distress (Becerra et al., 2021; Fynn et al., 2022).

### 1.3 The present study

Available data on the PAQ are therefore promising so far. However, there is a need to create and validate further language versions to facilitate alexithymia research across cultures. Our aim here was therefore to introduce the first French version of the PAQ, examine its psychometric properties, and in so doing, use the PAQ to further establish the nature of the alexithymia construct in the French-speaking context. Specifically, we examined the PAQ in a French-speaking Belgian sample. We examined its factor structure and reliability, and the nature of its relationships with other measures of alexithymia, emotion regulation, and psychopathology symptoms. To further understand the alexithymia construct at the facet level, we also used latent profile analysis to examine how different combinations of alexithymia facets (i.e., alexithymia profiles) may relate to psychopathology symptoms.

## 2 Method

### 2.1 Participants

Our sample comprised 481 participants (85% female) living in Belgium, with a mean age 24.09 ± 9.31 (range from 18 to 69). The sample was 86.3% students, 11.2% working professionals, and 2.5% people that were unemployed or retired. The highest education level completed for 81.7% was undergraduate university, and for 11.3% was graduate level university. In terms of relationship status, 60.9% of the sample was single. All participants completed an online survey (limesurvey) that was advertised on internet social media platforms, as well as through announcements at a French-speaking Belgian university. The study received approval from the local ethical committee (ULB: 1572/2023).

### 2.2 Materials

All psychometric tools were administered in their French language forms. We administered the PAQ to all participants, and a subset of these (*n* = 393) also completed other measures of alexithymia, emotion regulation, and psychopathology symptoms. All descriptive statistics and reliability estimates are provided in Table 1.

**Table 1:** Descriptive statistics and reliability coefficients for the administered measures.

**Variable**	**α**	**ϖ*_*t*_***	***n***	**Mean**	**SD**	**Min**	**Max**
**PAQ**							
**Subscales**							
N-DIF	0.80	0.80	481	15.48	6.16	4	28
P-DIF	0.82	0.83		10.93	5.39	4	28
N-DDF	0.88	0.88		16.46	6.88	4	28
P-DDF	0.82	0.83		12.49	5.71	4	28
G-EOT	0.92	0.93		22.81	11.88	8	56
**Composites**							
N-DAF	0.90	0.90		31.95	12.19	8	56
P-DAF	0.90	0.90		23.42	10.52	8	56
G-DAF	0.92	0.92		55.37	19.91	16	112
G-DIF	0.85	0.84		26.14	10.19	8	56
G-DDF	0.87	0.87		28.73	11.11	8	56
Total score	0.94	0.96		78.17	28.41	24	154
**ERQ**							
Cognitive reappraisal	0.75	0.72	446	26.61	7.45	6	42
Expressive suppression	0.80	0.84		15.37	6.20	4	28
**DASS-21**							
Depression	0.90	0.91	423	7.33	5.82	0	21
Anxiety	0.81	0.82		7.16	5.01	0	21
Stress	0.83	0.83		8.38	4.83	0	21
Total score	0.93	0.93		22.88	13.94	0	59
**DERS-SF**							
Total	0.87	0.89	434	45.76	12.55	21	84
Awareness	0.76	0.80		6.94	2.72	3	15
Clarity	0.72	0.74		7.07	2.78	3	14
Goals	0.91	0.90		8.24	2.92	3	15
Impulse	0.90	0.90		7.39	2.75	3	15
Strategy	0.78	0.79		8.01	2.97	3	15
Non-acceptance	0.85	0.86		8.10	2.69	3	15
**TAS-20**							
Total score	0.83	0.84	373	50.01	12.70	21	80
DIF	0.81	0.77		18.61	6.48	7	35
DDF	0.81	0.79		14.70	5.17	5	25
EOT	0.49	0.51		16.71	4.24	8	31

PAQ, Perth Alexithymia Questionnaire; N-DIF, Negative-Difficulties Identifying Feelings; P-DIF, Positive-Difficulties Identifying Feelings; N-DIF, Negative-Difficulties Describing Feelings; P-DDF, Positive-Difficulties Describing Feelings; G-EOT, General-Externally Oriented Thinking; N-DAF, Negative-Difficulties Appraising Feeling; P-DAF, Positive-Difficulties Appraising Feeling; G-DAF, General-Difficulties Appraising Feeling; G-DIF, General-Difficulties Identifying Feelings; G-DDF, general-Difficulties describing Feelings; ERQ, Emotion Regulation Questionnaire; DERS-18, Difficulties with Emotion Regulation Scale-18; TAS-2, Toronto Alexithymia Scale; DIF, Difficulties Identifying Feelings; DDF, Difficulties Describing Feelings; EOT, Externally Oriented Thinking; α, Cronbach alpha; ϖ*t*, Mc Donald Omega total.

#### 2.2.1 Perth alexithymia questionnaire (PAQ)

As noted earlier, the PAQ (Preece et al., 2018) is a 24-item questionnaire scored on a 7-point Likert scale, ranging from 1 (strongly disagree) to 7 (strongly agree). Higher scores indicate higher alexithymia. A total score as well as five subscale scores are designed to be derived: N-DIF, P-DIF, N-DDF, P-DDF, and G-EOT. These subscales can also be combined into various theoretically meaningful composites. N-DIF and P-DIF scores can be combined into a General-Difficulty Identifying Feelings (G-DIF) composite, and the N-DDF and P-DDF scores into a General-Difficulty Describing Feelings (G-DDF) composite, as non-valence specific markers of those alexithymia facets. Because DIF and DDF are both conceptually closely linked as reflecting the appraisal stage of emotion processing, the DIF and DDF subscales can also be combined into Negative-Difficulty Appraising Feelings (N-DAF), Positive-Difficulty Appraising Feelings (P-DAF), and General-Difficulty Appraising Feelings (G-DAF) composites. All items can also be summed into a total score as an overall marker of alexithymia.

##### 2.2.1.1 Translation process

An initial translation of the PAQ items from English to French was conducted and refined by members of the authorship team fluent in both languages. These French items were then back translated into English by an external NAATI approved translator. Minor refinements to some of the French items were made by the authorship team based on this back-translation, resulting in the final French version of the PAQ administered in this study. The French PAQ (Luminet et al., 2021c) and its scoring instructions are publicly available at https://doi.org/10.31234/osf.io/r7dft.

#### 2.2.2 Toronto alexithymia scale-20 (TAS-20)

The TAS-20 (Bagby et al., 1994) is a 20 items scale scored on a 5-point Likert scale. Higher scores indicate higher alexithymia. As noted by the developers, the TAS-20 was originally designed only to provide a total score as an overall marker of alexithymia (Bagby et al., 2007), though subscale scores for the DIF, DDF, and EOT facets are also commonly extracted in the field given high interest in the multidimensional nature of alexithymia (Luminet and Nielson, 2024). In the interest of completeness, in this paper we report both TAS-20 total and subscale scores. Similar to past work, in our sample, the total score, DIF and DDF subscale scores had acceptable internal consistency reliability, but the EOT subscale score did not.

#### 2.2.3 Emotion regulation questionnaire (ERQ)

The ERQ (Gross and John, 2003) is a 10-item self-report measure focused on emotion regulation in terms of frequency of regulation strategy use and rated on a 7-point Likert scale. It measures habitual use of two common reliable emotion regulation strategies, cognitive reappraisal (6 items) and expressive suppression (4 items). Separate scale scores are derived for each strategy, with higher scores indicating more use of that strategy. Cognitive reappraisal (i.e., changing the way you are thinking about a situation to change its emotional impact) is usually associated with good wellbeing outcomes, and expressive suppression (i.e., inhibiting behavioral expression of an emotion) is usually associated with poor wellbeing outcomes^1^ (Gross and John, 2003). As such, a profile of low cognitive reappraisal and high expressive suppression indicates emotion regulation difficulties. The ERQ has demonstrated good validity and reliability (Gross and John, 2003) and good internal consistency in our sample.

#### 2.2.4 Difficulties in emotion regulation scale-short form (DERS-SF)

The DERS-SF (Kaufman et al., 2016) is an 18-item self-report measure of overall difficulties in emotional functioning and regulation, with 18-items and six sub-scales designed to measure different facets of emotion regulation difficulties. Participants were asked to respond on a Likert scale from 1 (almost never) to 5 (almost always), with higher scores indicating more emotion regulation difficulties. The subscales include: (1) non-acceptance of emotional responses, (2) difficulties engaging in goal-oriented behaviors when experiencing negative emotions, (3) difficulties in controlling impulsive behaviors when experiencing negative emotions, (4) lack of emotional awareness, (5) lack of strategies to regulate emotions, and (6) lack of emotional clarity. The total and subscale scores exhibited good internal consistency in our sample.

#### 2.2.5 Depression anxiety stress scales-21 (DASS-21)

The DASS-21 (Lovibond and Lovibond, 1995) is a 21-item self-report measure scored on a 4-point Likert scale, with higher scores indicating more severe symptoms. It measures depression, anxiety and stress symptoms experienced during the last week. A total score can also be derived, as an overall marker of psychological distress. The DASS-21 demonstrates good validity and reliability (Bond and Wickham, 2023) and the total and subscale scores exhibit good internal consistency in our sample.

### 2.3 Statistical procedures

Descriptive statistics were calculated for the PAQ and all administered measures.

#### 2.3.1 Factor structure

Confirmatory Factor analyses of the PAQ were performed using Maximum Likelihood with Satorra-Bentler robust standard errors estimations (Satorra and Bentler, 1994) and Stata software (STATA 17, Stata Corp). Model fit was assessed using the criteria of Hu and Bentler (1999), based on several fit index values: the Comparative Fit Index (CFI: excellent fit: ≥ 0.95, acceptable fit: ≥ 0.90), the Root Mean Square Error of Approximation (RMSEA; excellent fit: ≤ 0.06, acceptable fit: ≤ 0.08), and the Standardized Root Mean Square Residual (SRMR; excellent fit: ≤ 0.06, acceptable fit: ≤ 0.08). To compare models whilst accounting for parsimony, we also reported the Akaike Information Criterion (AIC). Item factor loadings of 0.40 or more were considered as meaningful loadings.

**Figure 1:**
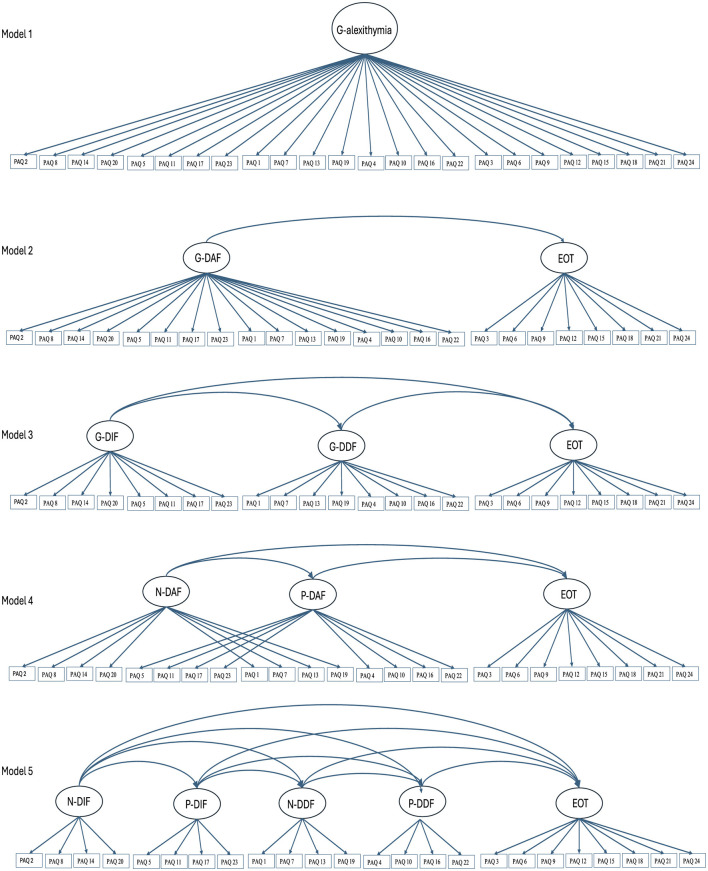
Perth alexithymia questionnaire lower-order factor structures.

Similar to past PAQ work, we estimated 5 theoretically informed lower-order models (see Figure 1). Model 1 was a unidimensional model with a general alexithymia factor. Model 2 was a two-factor correlated model, comprised of “General-Difficulty Appraising Feelings” (G-DAF) and “General-External Oriented Thinking” (G-EOT) factors; it therefore differentiated between the attention and appraisal stages of emotion processing, but did not differentiate between DIF and DDF, nor between the different valence domains. Model 3 was a three-factor correlated model, with “General-Difficulty Identifying Feelings” (G-DIF), “General-Difficulty Describing Feelings” (G-DDF) and “General-External Oriented Thinking” (G-EOT) factors; it therefore differentiated the three facets of alexithymia but did not account for the different valence domains. Model 4 was also a three-factor correlated model but comprised instead of “Negative-Difficulty Appraising Feelings” (N-DAF), “Positive-Difficulty Appraising Feelings” (P-DAF), and “General-External Oriented Thinking” (G-EOT) factors; it therefore accounted for valence in the appraisal domain but did not split DIF and DDF. Model 5 was a five-factor correlated model, comprised of “Negative-Difficulty Identifying Feelings” (N-DIF), “Positive-Difficulty Identifying Feelings” (P-DIF), “Negative-Difficulty Describing Feelings” (N-DDF), “Positive-Difficulty Describing Feelings” (P-DDF) and “General Externally Oriented Thinking” (G-EOT) factors; it therefore reflected the intended five subscale structure of the PAQ, differentiating between the DIF, DDF, and EOT facets of alexithymia, and accounting for the negative and positive valence domains.

Additionally, we examined bifactor versions of the tenable first-order models. That is, we explored models where a “general” factor was included alongside the “narrow” subscale factors (Markon, 2019). The tenability of a general factor helps to establish whether a total score should be used. Indeed, a frequent critic in clinical research is the use of both total and subscale scores from measures without strong measurement arguments to do so (Reise et al., 2013). The recommended reliability estimate for bifactor models is omega hierarchical (Gignac, 2014; Dunn et al., 2014). We used the omega Sem function of psych R package with Lavaan (Revelle, 2013). An EFA with oblimin rotation is first run to apply the Schmid-Leiman transformation allowing to estimate loadings for the general factor. A CFA is then run on the Schmid-Leiman solution and omega hierarchical is extracted. Items' subdimensions are not defined a-priori. If the resulting model aligned items correctly in a meaningful dimension, this is another strong support for the reliability of the measurement structure, especially because bifactor models tend to evidence better fit than other models even when misspecified (Gignac, 2016).

#### 2.3.2 Concurrent validity

We calculated Pearson correlations between the PAQ, TAS-20, DERS-SF, ERQ, and DASS-21 to evaluate the concurrent validity of the PAQ. Because the PAQ and TAS-20 are both designed as measures of alexithymia, we expected them to correlate highly. Similarly, because alexithymia conceptually should impair emotion regulation processes (Mehta et al., 2024; Panayiotou et al., 2021; Preece et al., 2023), we expected PAQ scores to be significantly associated with more emotion regulation difficulties on the DERS-SF and higher use of expressive suppression and lower use of cognitive reappraisal on the ERQ. Because alexithymia is a risk factor for psychopathology, we similarly expected PAQ scores to relate to higher depression, anxiety, and stress symptoms.

#### 2.3.3 Discriminant validity

Whilst PAQ scores should be correlated with psychopathology symptoms, alexithymia should nonetheless be a separable construct from psychopathology symptoms (i.e., to be valid, the PAQ should measure alexithymia, not people's current levels of distress). To test discriminant validity in this respect and following recent recommendations (Cheung et al., 2024) we used structural equation modeling. First, two models were fitted: Model 1, which included distinct latent factors for PAQ and DASS-21 with freely estimated covariance, and Model 2, which consisted of a single latent factor encompassing all dimensions of both measures. The fit of these models was compared using a Likelihood Ratio (LR) test. Second, Model 1 was compared to an alternative version of the same model (Model 3), in which the latent covariance was constrained to 1. Finally, we computed the heterotrait-monotrait ratio (HTMT) of correlations (Henseler, 2017; Henseler et al., 2015; Rönkkö and Cho, 2022). We used the modified version of HTMT based on geometric means (HTMT2; Roemer et al., 2021). A ratio of ≥ 0.85 indicates a violation of discriminant validity. We use the SemTools R package (Jorgensen et al., 2018).

#### 2.3.4 Latent profile analysis

To further explore the nature of alexithymia presentations and their clinical relevance, we conducted a latent profile analysis (LPA) to explore profile combinations of alexithymia facets (PAQ scores) and depression, anxiety, stress symptoms (DASS-21). LPA is a method that allows the extraction of different subgroups (or subtypes or profiles) within the sample, who exhibit a similar pattern of scores across a set of variables. We used the TidyLPA package in *R* software (Rosenberg et al., 2019). *Z*-scores of all the PAQ and DASS-21 subscales were entered as the variables. Using the default model parameters of TidyLPA (i.e., Model 1: equal variances and covariances fixed to 0), solutions for 1 to 12 profiles were calculated and compared. The best solution was judged based on commonly used fit index values and classification statistics (Weller et al., 2020): the Bayesian Information Criterion (BIC), the Sample Size adjusted Bayesian Information Criterion (SABIC), Akaike Information Criterion (AIC), Approximate Weight of Evidence Criterion (AWE), Classification Likelihood Criterion (CLC), and Kullback Information Criterion (KIC). Lower fit index values indicate better fit. We considered also Bootstrapped Likelihood Ratio Tests (BLRT) to determine if an increase in the number of profiles by 1 significantly improved model fit (*p* < 0.05 indicating that the more complex solution is superior). We also looked at classification diagnostic statistics such as Entropy (>0.80 being acceptable), and minimum average latent posterior probabilities (Min_prob). The best model was selected based on a combination of theoretical interpretability, BIC (often found to be the best single indicator amongst fit indices; Nylund et al., 2007), and the acceptability criteria of the other fit or classification indices (AIC, BIC, AWE, KIK and SABIC, entropy). To be tenable, a profile solution also needed to have at least 5% of the sample in its smallest profile, in order to avoid rare profiles that are unlikely to replicate. In interpreting z-score levels within the LPA profiles, scores < 0.5 SD above or below the sample mean were considered as being in the average range; scores between 0.5 SD and 1 SD above or below the mean were considered moderately high or moderately low; and scores >1 SD above or below the mean were considered high or low scores (Preece et al., 2024a).

## 3 Results

Reliability coefficients, sample size, means, standard deviations, and score ranges are provided in Table 1 for the PAQ and other administered measures.

### 3.1 Factor structure

All CFA indices (see Table 2) indicated that, in terms of the lower-order structure of the PAQ, it was best represented by Model 5 (the five-factor correlated model), with Model 4 (the three-factor correlated model with valence-specific appraisal factors) also being tenable with acceptable fit in all indices.

**Table 2:** Goodness of fit indexes of PAQ models.

**Model**	**χ^2^-SB (df)**	**CFI-SB**	**TLI-SB**	**RMSEA-SB**	**SRMR**	**AIC**
Model 1	2,238.06 (252)	0.63	0.60	0.13	0.12	42,395.93
Model 2	1,388.46 (251)	0.79	0.77	0.97	0.86	41,234.37
Model 3	1,375.80 (249)	0.79	0.77	0.97	0.85	41,221.50
Model 4	626.67 (249)	0.93	0.92	0.56	0.50	40,192.95
Model 5	554.02 (242)	0.94	0.93	0.52	0.46	40,109.13
**Higher order bi-factor model**						
Bi-factor model based on model 4	551.58 (228)	0.94	0.93	0.54	0.40	4,135.41

χ^2^-SB, Chi-square_satora-Bentler; CFI-SB, Comparative Fit Index-Satora-Bentler; TLI-SB, Tucker Lewis index-Satora-Bentler; RMSEA-SB, root mean square error of approximation-Satora-Bentler; SRMR, standardized root mean residual; AIC, Akaike information criterion.

All the lower-order models that did not account for valence were a poor fit to the data. The unidimensional model (Model 1) was a poor fit to the data, highlighting that the PAQ was assessing a multidimensional construct. Splitting between the attention and appraisal components of alexithymia improved fit (Model 2), though fit index values were still poor. Splitting between DIF, DDF, and EOT (Model 3), further improved fit, but not to acceptable levels. The superiority of Model 4 over Model 3 suggests within the factor structure that it is more important to differentiate between the appraisal of negative and positive valences, than it is to differentiate between the DIF and DDF components of appraisal. Indeed, the DIF and DDF factors within each valence domain were highly correlated in Model 5. However, the fit index values were generally better for Model 5 than Model 4, highlighting that there is still some statistical value in separating DIF and DDF within each valence domain. AIC was in favor of Model 5 over Model 4, and the likelihood ratio test comparing both models was significant [χ^2^(7) = 87.92, *p* ≤ 0.001]. All factor loadings for Model 5 ranged from 0.60 to 0.86 (Table 3). Thus, the lower-order structure of the PAQ seemed to be best represented by the 5 intended subscale factors.

**Table 3:** Factor loadings for the best fitting lower-order and bifactor models of the PAQ.

**Item**	**Item content**		**Model 5 lower-order model—factor loadings**	**Model 4-bifactor model—factor loadings**
2	N-DIF	When I'm feeling bad, I can't tell whether I'm sad, angry, or scared.	0.67	0.46 (0.44)
8			When I'm feeling bad, I can't make sense of those feelings.	0.78	0.44 (0.62)
14			When I'm feeling bad, I get confused about what emotion it is.	0.78	0.56 (0.48)
20			When I'm feeling bad, I'm puzzled by those feelings.	0.60	0.42 (0.37)
1	N-DDF	When I'm feeling bad (feeling an unpleasant emotion), I can't find the right words to describe those feelings.	0.80	0.61 (0.52)
7			When I'm feeling bad, I can't talk about those feelings in much depth or detail.	0.76	0.42 (0.62)
13			When something bad happens, it's hard for me to put into words how I'm feeling.	0.86	0.58 (0.63)
19			When I'm feeling bad, if I try to describe how I'm feeling I don't know what to say.	0.81	0.52 (0.61)
5	P-DIF	When I'm feeling good, I can't tell whether I'm happy, excited, or amused.	0.64	0.40 (0.48)
11			When I'm feeling good, I can't make sense of those feelings.	0.77	0.44 (0.62)
17			When I'm feeling good, I get confused about what emotion it is.	0.86	0.70 (0.54)
23			When I'm feeling good, I'm puzzled by those feelings.	0.70	0.57 (0.42)
4	P-DDF	When I'm feeling good (feeling a pleasant emotion), I can't find the right words to describe those feelings.	0.65	0.35 (0.53)
10			When I'm feeling good, I can't talk about those feelings in much depth or detail.	0.70	0.29 (0.64)
16			When something good happens, it's hard for me to put into words how I'm feeling.	0.86	0.56 (0.64)
22			When I'm feeling good, if I try to describe how I'm feeling I don't know what to say.	0.78	0.41 (0.65)
3	G-EOT	I tend to ignore how I feel.	0.75	0.58 (0.49)
6			I prefer to just let my feelings happen in the background, rather than focus on them.	0.81	0.56 (0.59)
9			I don't pay attention to my emotions.	0.85	0.69 (0.53)
12			Usually, I try to avoid thinking about what I'm feeling.	0.80	0.45 (0.67)
15			I prefer to focus on things I can actually see or touch, rather than my emotions.	0.74	0.51 (0.53)
18			I don't try to be “in touch” with my emotions. / Je n'essaye pas d'être en contact avec mes émotions.	0.80	0.53 (0.59)
21			It's not important for me to know what I'm feeling. / Ce n'est pas important pour moi de savoir ce que je ressens.	0.72	0.55 (0.46)
24			It's strange for me to think about my emotions. / C'est bizarre pour moi de penser à mes émotions.	0.75	0.49 (0.56)

For the bifactor model, factor loadings inside the brackets are loadings on the broad “general alexithymia” factor, and factor loadings outside the brackets are loadings on the narrow one. N-DIF, Negative-Difficulties Identifying Feelings; P-DIF, Positive-Difficulties Identifying Feelings; N-DIF, Negative-Difficulties Describing Feelings; P-DDF, Positive-Difficulties Describing Feelings; G-EOT, General-Externally Oriented Thinking. Model 5: Lower-Order Correlated Model—Factor Loadings.

Because Model 5 and Model 4 were both tenable, we used these as the bases for our bifactor model testing, testing the tenability of a general factor. Model 5's bifactor variant did not converge in our modeling, perhaps because of the added complexity of the general factor loadings alongside the high correlations between the DIF and DDF factors. Model 4's bifactor variant (i.e., comprised of a “general alexithymia” factor and narrow “N-DAF,” “P-DAF,” and “G-EOT” factors) did converge, and displayed strong levels of fit (see Table 2). The common explained variance of the general factor in this model was above the 0.65 threshold meaning that 73 % of the total scores variance could be attributed to the individual differences on the general factor (ω_h_ = 0.72). The omega for the total reliability of the scale, including the “general alexithymia” factor and the narrow factors (i.e., N-DAF, P-DAF, and G-EOT), was also above the 0.80 threshold (ω_t_ = 0.96) meaning that 96% of the variance of the total PAQ score is due to a combination of the general factor and the narrow factors. By extension, the variance explained by the general factor independently of the subfactors, was 0.75 (ω_h/_ω_t_), the variance explained by subdimensions independently of the general factor was 0.46 for G-EOT, 0.41 for N-DAF and 0.39 for P-DAF. Factor loadings for each item indicated meaningful loadings on the general factor and their designated narrow factor (see Table 3). Thus, our bifactor modeling indicated good support for the tenability of a “general alexithymia” factor in the PAQ, and therefore the summing of all items into a total score.

### 3.2 Internal consistency reliability

Omega and Alpha reliabilities for the PAQ total score (i.e., general alexithymia) were excellent (α = 0.94; ω_t_ = 0.96) and Omega and Alpha reliabilities for all the PAQ subscale scores and other composite scores were also high, ranging from 0.80 to 0.93 (see Table 1).

Pearson's correlations between all PAQ scores are provided in Table 4. All PAQ subscales were significantly positively correlated with each other, ranging from 0.40 to 0.80. The strongest correlations were between the DIF and DDF facets within the same valence domain (*r* = 0.75 between N-DIF and N-DDF and *r* = 0.80 between P-DIF and P-DDF), all other subscale correlations were moderate between 0.40 and 0.51.

**Table 4:** Pearson correlations between Perth alexithymia questionnaire scores.

**Variables**		**(1)**	**(2)**	**(3)**	**(4)**	**(5)**	**(6)**	**(7)**	**(8)**	**(9)**	**(1)**
Subscale factor scores	(1) N-DIF	1									
	(2) P-DIF	0.49	1								
	(3) N-DDF	0.75	0.4	1							
	(4) P-DDF	0.51	0.8	0.51	1						
	(5) G-EOT	0.4	0.48	0.5	0.5	1					
Composite scores	(6) G-DIF	0.88	0.84	0.68	0.75	0.51	1				
	(7) G-DDF	0.73	0.67	0.9	0.84	0.58	0.81	1			
	(8) N-DAF	0.93	0.47	0.94	0.55	0.48	0.83	0.88	1		
	(9) P-DAF	0.52	0.95	0.48	0.95	0.52	0.84	0.8	0.54	1	
	(10) G-DAF	0.84	0.79	0.83	0.84	0.57	0.95	0.96	0.9	0.86	1
	(11) PAQ-TOTAL	0.76	0.75	0.79	0.8	0.82	0.88	0.91	0.83	0.82	0.94

All Pearson's are significant at p ≤ 0.1. N-DIF, Negative-Difficulties Identifying Feelings; P-DIF, Positive-Difficulties Identifying Feelings; N-DIF, Negative-Difficulties Describing Feelings; P-DDF, Positive-Difficulties Describing Feelings; G-EOT, General-Externally Oriented Thinking; G-DIF, General-Difficulties Identifying Feelings; G-DDF, General-Difficulties Describing Feelings; N-DAF, Negative-Difficulties Appraising Feeling; P-DAF, Positive-Difficulties Appraising Feeling; G-DAF, General-Difficulties Appraising Feeling; PAQ Total, Perth Alexithymia Questionnaire-Total scores.

### 3.3 Concurrent validity

Correlations between the PAQ and other measures are displayed in Table 5. The total scores of the PAQ and TAS-20 were highly correlated (0.80). Across the PAQ and TAS-20 facet scores, there appeared to be a high level of concordance, with DIF scores correlating most highly with each other, and DDF scores correlating most highly with each other. The exception to this pattern was the EOT scores, where the PAQ G-EOT score did correlate significantly with the TAS-20 EOT score (0.45), but correlated most highly with TAS-20 DDF (0.55); this comparison though is likely impacted by the low reliability of the TAS-20 EOT subscale score (α = 0.48), with the high level of error variance making high correlations with that score difficult. In support of the PAQ's concurrent validity, PAQ scores were generally significantly correlated with higher emotion regulation difficulties (i.e., total score and subscale scores) on the DERS-SF, except for G-EOT which was uncorrelated with the DERS-SF non-acceptance subscale. More use of expressive suppression and less use of cognitive reappraisal (ERQ), as well as higher levels of depression, anxiety, and stress (DASS-21), were also significantly related to PAQ scores (see Table 5).

**Table 5:** Pearson correlations between the Perth alexithymia questionnaire and other measures of alexithymia, emotion regulation, and psychopathology symptoms.

**PAQ**	**DERS-SF**						**TAS-20**			**DASS-21**					**ERQ**	
	**Awareness**	**Clarity**	**Goals**	**Impulse**	**Non acceptance**	**Total**	**Total**	**DIF**	**DDF**	**EOT**	**Depression**	**Anxiety**	**Stress**	**Total**	**Cognitive reappraisal**	**Expressive suppression**
**PAQ subscales**																
N-DIF	0.33^***^	0.66^***^	0.46^***^	0.46^***^	0.43^***^	0.62^***^	0.72^***^	0.73^***^	0.63^***^	0.29^***^	0.38^***^	0.41^***^	0.42^***^	0.45^***^	−0.19^***^	0.43^***^
P-DIF	0.31^***^	0.56^***^	0.29^***^	0.30^***^	0.25^***^	0.43^***^	0.57^***^	0.56^***^	0.47^***^	0.30^***^	0.40^***^	0.30^***^	0.31^***^	0.38^***^	−0.18^***^	0.39^***^
N-DDF	0.39^***^	0.56^***^	0.32^***^	0.30^***^	0.27^***^	0.48^***^	0.73^***^	0.60^***^	0.78^***^	0.33^***^	0.30^***^	0.30^***^	0.24^***^	0.32^***^	−0.16^**^	0.52^***^
P-DDF	0.33^***^	0.52^***^	0.28^***^	0.27^***^	0.20^***^	0.41^***^	0.59^***^	0.53^***^	0.55^***^	0.29^***^	0.39^***^	0.27^***^	0.24^***^	0.34^***^	−0.18^***^	0.42^***^
G-EOT	0.68^***^	0.52^***^	0.18^***^	0.18^***^	0.09	0.39^***^	0.58^***^	0.39^***^	0.54^***^	0.47^***^	0.35^***^	0.21^***^	0.11^*^	0.26^***^	−0.07	0.63^***^
**PAQ composites**																
P-DAF	0.34^***^	0.57^***^	0.30^***^	0.30^***^	0.24^***^	0.44^***^	0.62^***^	0.57^***^	0.54^***^	0.31^***^	0.42^***^	0.30^***^	0.29^***^	0.38^***^	−0.19^***^	0.43^***^
N-DAF	0.39^***^	0.65^***^	0.42^***^	0.40^***^	0.37^***^	0.59^***^	0.78^***^	0.70^***^	0.76^***^	0.34^***^	0.36^***^	0.38^***^	0.35^***^	0.41^***^	−0.19^***^	0.51^***^
G-DIF	0.37^***^	0.71^***^	0.44^***^	0.44^***^	0.40^***^	0.62^***^	0.76^***^	0.75^***^	0.64^***^	0.34^***^	0.45^***^	0.42^***^	0.42^***^	0.48^***^	−0.22^***^	0.47^***^
P-DIF	0.31^***^	0.56^***^	0.29^***^	0.30^***^	0.25^***^	0.43^***^	0.57^***^	0.56^***^	0.47^***^	0.30^***^	0.40^***^	0.30^***^	0.31^***^	0.38^***^	−0.18^***^	0.39^***^
Total score	0.57^***^	0.71^***^	0.37^***^	0.36^***^	0.29^***^	0.58^***^	0.80^***^	0.68^***^	0.75^***^	0.45^***^	0.45^***^	0.36^***^	0.30^***^	0.42^***^	−0.18^***^	0.64^***^

N-DIF, Negative-Difficulties Identifying Feelings; P-DIF, Positive-Difficulties Identifying Feelings; N-DDF, Negative-Difficulties Describing Feelings; P-DDF, Positive-Difficulties Describing Feelings; G-EOT, General-Externally Oriented Thinking; P-DAF, Positive-Difficulties Appraising Feeling; N-DAF, Negative-Difficulties Appraising Feeling; G-DIF, General-Difficulties Identifying Feelings; G-DDF, General-Difficulties Describing Feelings; G-DAF, General-Difficulties Appraising Feeling; PAQ Total, Perth Alexithymia Questionnaire-Total scores; DERS-SF, Difficulties with Emotion Regulation Scale-Short Form; TAS-20, Toronto Alexithymia Scale-20; DASS-21, Depression Anxiety Stress Scale-21; ERQ, Emotion Regulation Questionnaire. ^*^p ≤ 0.05, ^**^p ≤ 0.01, ^***^p ≤ 0.001.

### 3.4 Discriminant validity

To evaluate the discriminant validity of the PAQ against distress, we estimated the HTMT2 criterion considering the PAQ and DASS-21 constructs, composed by their respective subfactors. The value of the HTMT2 was lower than the 0.85 threshold (HTMT2 = 0.50), thus indicating that the PAQ exhibited good discriminant validity against the DASS-21. Furthermore, a comparison of two competing models (refer to the statistics section) revealed that the model with a single factor demonstrated a poorer fit compared to the model with distinct latent factors for the PAQ and DASS-21 [Likelihood Ratio test: χ^2^(1) = 906.72, p ≤ 0.001]. Similarly, when comparing a two-latent-factor model with freely estimated covariance to the same model with covariance constrained to 1, the latter exhibited a significantly worse fit [Likelihood Ratio test: χ^2^(1) = 79.52, p ≤ 0.001]. These three analyses support the discriminant validity of the PAQ.

### 3.5 Latent profile analysis

Our LPA of alexithymia and psychopathology profiles indicated that, of the tenable profiles (containing all profiles with at least 5% of the sample), an eight-profile solution best summarized the data (see Supplementary material for the fit of the 12 models estimated). The eight-profile solution had the best performance on BIC and performed well across the other indicators (e.g., entropy = 0.86; average minimum probability for accurately predicting class membership for individuals = 0.84), producing a highly theoretically interpretable solution (see Supplementary Table S1 for all model fit indices).

**Figure 2:**
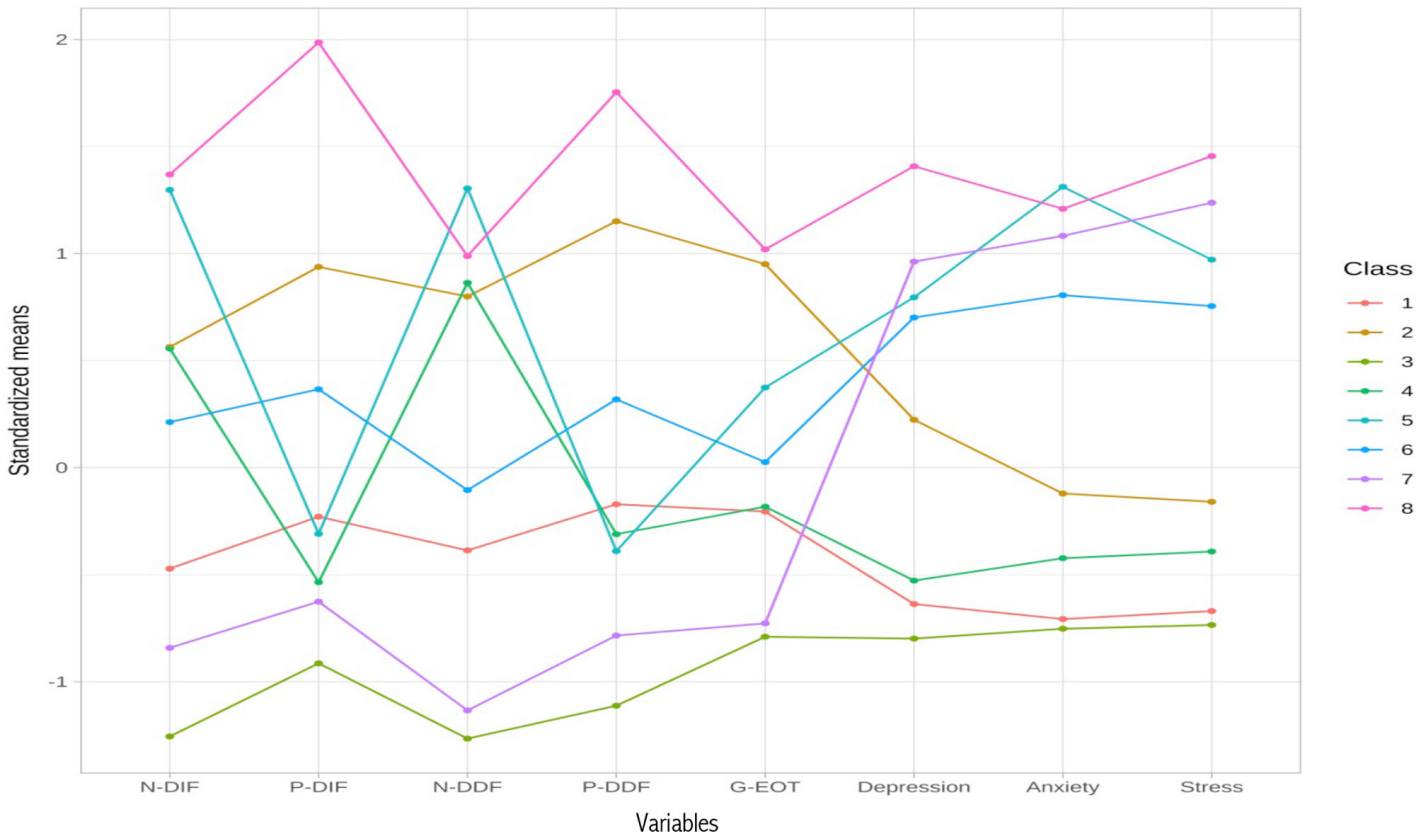
Latent profiles of alexithymia (PAQ scores) and psychopathology symptoms (DASS-21 scores).

In terms of clinical relevance, four profiles had elevated levels of psychopathology symptoms (Profiles 5, 6, 7, and 8) and four profiles had average or low levels of psychopathology symptoms (Profiles 1, 2, 3, and 4). Levels of depression, anxiety, and stress were generally similar levels within each profile (i.e., no unique profile for depression vs. anxiety). Latent profiles are displayed in Figure 2.

#### 3.5.1 Elevated psychopathology profiles

Amongst those profiles with heightened psychopathology symptoms, two had elevated alexithymia and two did not. Profile 8 (*n* = 72) had amongst the highest psychopathology levels, and also exhibited high levels of alexithymia across all facets and valence domains. In contrast, Profile 5 (*n* = 74) had similar psychopathology levels, but its alexithymia profile was characterized only by difficulties appraising negative feelings (i.e., N-DIF, N-DDF), with average scores in the appraisal of positive feelings (i.e., P-DIF, P-DDF) and externally oriented thinking. Profile 7 (*n* = 20) had generally high psychopathology symptoms but low alexithymia on all domains, and Profile 6 (*n* = 46) had moderately high psychopathology symptoms but average alexithymia on all domains.

#### 3.5.2 Non-elevated psychopathology profiles

Of the four profiles with generally average psychopathology symptoms, two had elevated alexithymia and two did not. Profile 3 (*n* = 30) had amongst the lowest psychopathology symptoms and amongst the lowest alexithymia across all facets and valence domains. Profile 1 (*n* = 63) had similarly low psychopathology levels to Profile 3 but had alexithymia in the average range for all domains. Of the two alexithymia profiles, Profile 2 (*n* = 26) had elevated alexithymia in all facets and valence domains, whilst Profile 4 (*n* = 92) was similar to Profile 5 in its alexithymia profile—exhibiting difficulties only in the appraisal of negative (not positive) emotions, also with no difficulties in EOT.

## 4 Discussion

The aim of our study was to introduce the first French language version of the PAQ, examine its psychometric properties, and in so doing, explore the nature of the alexithymia construct. Overall, our results suggest that the French PAQ has strong validity and reliability as a measure of the multidimensional alexithymia construct.

### 4.1 The latent structure of alexithymia

Similar to findings with the PAQ in other language forms (Cai et al., 2024; Becerra et al., 2021; Larionow et al., 2022), in our CFAs we found that the French PAQ's lower-order factor structure was well represented by five factors, corresponding to the intended 5 subscales structure (i.e., N-DIF, P-DIF, N-DDF, P-DDF, and G-EOT). All items loaded well on their intended factors, and this structure therefore emphasizes the multidimensional nature of alexithymia, with value in separating between the DIF, DDF, and EOT facets of alexithymia, and between the negative and positive valence domains. The close connection we observed between DIF and DDF (and models that combine them also being highly tenable) is in line with the predictions of the attention-appraisal model of alexithymia (Preece et al., 2017). It is also consistent with past work that has formulated DIF and DDF as closely linked (e.g., Taylor et al., 1999). Like past data in other languages (Chan et al., 2023; Fynn et al., 2022), the importance of valence within the PAQ's structure was particularly prominent, with those models that did not differentiate between negative and positive valence at the appraisal stage displaying poor fit. We also found support in a bifactor solution for a strong general factor within the alexithymia construct, with items from all the alexithymia subdomains (and valence domains) loading on this factor (Preece et al., 2018). As such, our findings confirm that difficulties processing both negative and positive emotions appear to be prominent features of the alexithymia construct (Fantini-Hauwel et al., 2015; Preece et al., 2024a; Botella et al., 2015). Moving forward, the measurement of both valence domains should therefore help to enable more fine-grained alexithymia assessments. The PAQ is presently the only alexithymia measure that assesses processing difficulties for both negative and positive emotions, hence this appears to be a novel strength of the measure and advancement for the field.

Like past PAQ studies (Preece et al., 2018; Becerra et al., 2021; Larionow et al., 2022), we also found that all PAQ subscale and composite scores exhibited good levels of reliability, including the EOT facet score. There is therefore good support for their use. Previous studies using the TAS-20, including its French version (Loas et al., 2001), reported low reliability for the EOT dimension, leading to speculation that this facet of alexithymia may be inherently difficult to capture reliably (Gignac et al., 2007; Taylor et al., 2003).

Promisingly, our findings reinforce that with the PAQ, all facets of alexithymia appear to be able to be assessed robustly, including EOT. Given that the alexithymia field is increasingly interested in facet-level assessment of the multidimensional alexithymia construct—with a recent review highlighting this as one of the major future frontiers for the field (Luminet and Nielson, 2024)—the presence of reliable facet scores within the PAQ could help to enable such work.

### 4.2 Relationships with other measures/constructs

Our findings further reinforce the clinical relevance of the alexithymia construct as assessed by the PAQ. Strong relationships found between the PAQ and TAS-20 reinforce that they do measure a similar alexithymia construct. PAQ scores, as expected, were also linked with poorer overall emotion regulation, more use of maladaptive emotion regulation strategies, and less use of adaptive emotion regulation strategies. Similarly, high PAQ scores were linked with more severe self-reported symptoms of depression, anxiety and stress. These findings are consistent with similar work in other language versions (Cai et al., 2024; Becerra et al., 2021; Larionow et al., 2022; Lashkari et al., 2021). Conceptually, these findings are consistent with the status of alexithymia as an important transdiagnostic risk factor for the development and maintenance of psychopathologies, like depression and anxiety disorders (Luminet et al., 2021a; Leweke et al., 2011; Preece et al., 2022). They also correspond with predictions from the attention-appraisal model of alexithymia (Preece and Gross, 2023) and process model of emotion regulation (Gross, 2015) that, because attention to and appraisal of emotions is important for informing emotion regulation decisions, alexithymia should impair emotion regulation processes. Importantly, our findings also show that the PAQ has good discriminant validity against markers of psychopathology. Since the early stages of the alexithymia field, researchers have been concerned about whether self-report measures of alexithymia are validly measuring the construct, rather than just general ill-being (Leising et al., 2009). Reassuringly, our results confirm that the French PAQ appears to perform well in this respect, supporting a vital property for confident use in clinical research and practice.

Moreover, in using the PAQ to further understand the nature of alexithymia, our LPA illustrated how different combinations of alexithymia facets may present together in meaningful alexithymia profiles. Of the eight profiles we extracted, four had elevated levels of alexithymia. The profile with amongst the highest alexithymic difficulties across all alexithymia facets and valence domains was also the profile that had amongst the highest psychopathology symptoms. However, two other profiles had valence-specific deficits in alexithymia, exhibiting difficulties only in the appraisal of negative (not positive) emotions. Our LPA findings in this respect are consistent with past LPA work with the English version of the PAQ (Preece et al., 2024a). Importantly, our LPA also illustrates that whilst, on average, alexithymia is linked with higher depression and anxiety, not all people with high alexithymia are currently experiencing depression and anxiety, just as not all people with high depression and anxiety symptoms also have high alexithymia. These results are consistent with the complex nature of mental illness, and that there are many factors that can contribute to psychopathology risk, not just alexithymia (Kotov et al., 2017).

Taken together, these LPA findings suggest there may be meaningful subtypes of alexithymia, with some people exhibiting processing deficits in both valence domains, and some only for negative emotions. These findings could have important implications for the design of personalized and targeted alexithymia interventions (Preece and Sikka, 2024). For example, they suggest that, for many people with high alexithymia, interventions will likely need to help them both focus attention more readily on emotions (i.e., EOT) and appraise those emotions in more accurate and detailed ways (i.e., DIF, DDF), doing this for both negative and positive emotions. For some people though, it is only the appraisal of negative emotions that is problematic, and thus that this should be a more targeted intervention focus for those individuals. Techniques such as emotion psychoeducation, mindfulness, and practice discussing emotions in the therapy room, may be helpful in these endeavors (Norman et al., 2019; Salles, 2023). Future work will be important to continue exploring the replicability of these alexithymia profiles and their implications for wellbeing and psychological health.

## 5 Limitations and future directions

There are some limitations of our study that will require further research. Our sample was primarily female and university students. Other language versions of the PAQ have shown similar performance across different age, gender, and education groups, as well as across clinical and non-clinical samples (Greene et al., 2020; Chan et al., 2023; Trimble et al., 2024; Mazidi et al., 2023), however, it remains to be seen whether this is the case for the French PAQ. In the same vein, latent profile analysis could consider potential differences regarding gender-specific profiles in future studies. Future work in other sample types, including larger representative general community samples and clinical samples will be important, as well as establishing test-retest reliability. Also, our markers of concurrent validity were all other self-report questionnaires, future work using behavioral or lab-based markers of relevant variables will be useful to further examine the predictive utility of PAQ scores.

## 6 Conclusions

Our data suggest that the PAQ, in its French language form, has strong validity and reliability. It measures a conceptually coherent, multidimensional alexithymia construct, allowing the robust assessment of alexithymia at different levels of abstraction (i.e., general and facet-level assessments). Difficulties processing both negative and positive emotions seem to be prominent parts of the alexithymia construct, and thus the novel capacity of the PAQ to assess both valence domains seems a meaningful contribution to the field. Added to this, our data support that the PAQ enables confident assessment of EOT, an aspect of alexithymia that has traditionally been difficult to robustly capture in the field. This opens new, more detailed avenues for alexithymia research, and our introduction of this French version of the PAQ should help enable such directions in French-speaking cultural contexts. Given that this is the first study of the French PAQ, future studies will be important to test the replicability and generalizability of our findings.

## Data Availability

The raw data supporting the conclusions of this article will be made available by the authors, without undue reservation.

## Footnotes

^1^If research typically indicates that expressive suppression is associated with poorer outcomes, it is important to note that under certain specific circumstances, expressive suppression may have beneficial effects. Since we did not manipulate contextual variables, our analysis remained at a more general level.
