# Supplementary material for: Exploring alexithymia with the French Perth alexithymia questionnaire: latent structure, profiles, and links with affective outcomes
Source: Front Psychol. 2025 Jun 17;16:1615612. doi: 10.3389/fpsyg.2025.1615612 (PMC12209315; doi:10.3389/fpsyg.2025.1615612)

Supplementary file

**Table S1**

*Fit indices of Latent Profile Analysis*

| Model | BIC | AIC | AWE | CLC | KIC | SABIC | Entropy | Min.prob | Min.n | BLRT_p |
| --- | --- | --- | --- | --- | --- | --- | --- | --- | --- | --- |
| 1 | 9692 | 9627 | 9835 | 9597 | 9646 | 9641 | 1.00 | 1.00 | 1.00 | - |
| 2 | 8864 | 8763 | 9089 | 8714 | 8791 | 8785 | 0.82 | 0.93 | 0.44 | 0.01 |
| 3 | 8655 | 8518 | 8961 | 8451 | 8555 | 8547 | 0.85 | 0.87 | 0.21 | 0.01 |
| 4 | 8422 | 8248 | 8809 | 8163 | 8294 | 8285 | 0.86 | 0.87 | 0.14 | 0.01 |
| 5 | 8366 | 8156 | 8835 | 8054 | 8211 | 8201 | 0.86 | 0.86 | 0.08 | 0.01 |
| 6 | 8274 | 8027 | 8824 | 7907 | 8091 | 8080 | 0.84 | 0.86 | 0.09 | 0.01 |
| 7 | 8262 | 7979 | 8894 | 7841 | 8052 | 8040 | 0.83 | 0.80 | 0.08 | 0.01 |
| 8 | 8211 | 7891 | 8924 | 7735 | 7973 | 7960 | 0.86 | 0.84 | 0.05 | 0.01 |
| 9 | 8239 | 7883 | 9034 | 7709 | 7974 | 7960 | 0.85 | 0.75 | 0.04 | 0.05 |
| 10 | 8230 | 7838 | 9106 | 7645 | 7938 | 7922 | 0.85 | 0.79 | 0.05 | 0.01 |
| 11 | 8206 | 7777 | 9164 | 7567 | 7886 | 7870 | 0.86 | 0.80 | 0.04 | 0.01 |
| 12 | 8282 | 7817 | 9321 | 7588 | 7935 | 7917 | 0.86 | 0.79 | 0.00 | 0.941 |

BIC = Bayesian Information Criterion, AIC = Akaike Information Criterion, AWE = Appropriate Weight of Evidence Criterion, CLC = Classification Likelihood Criterion, KIC = Kullback Information Criterion.,, SABIC = Sample Size adjusted Bayesian Information Criterion, Min.prob = Minimum average probability of accurately predicting class membership, Min.n = minimum class sample size, BLRT = Bootstrapped Likelihood Ratio Test p value.

**Table S2**

*Discriminant Validity : confirmatory factor analysis models*

Model 1


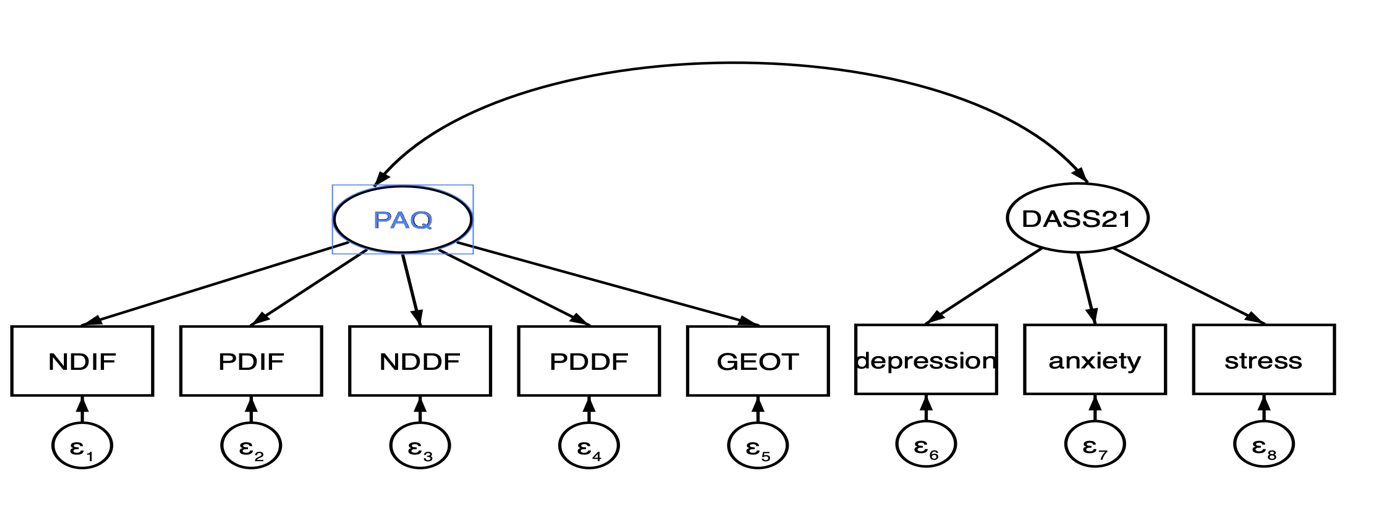


Model 2 :


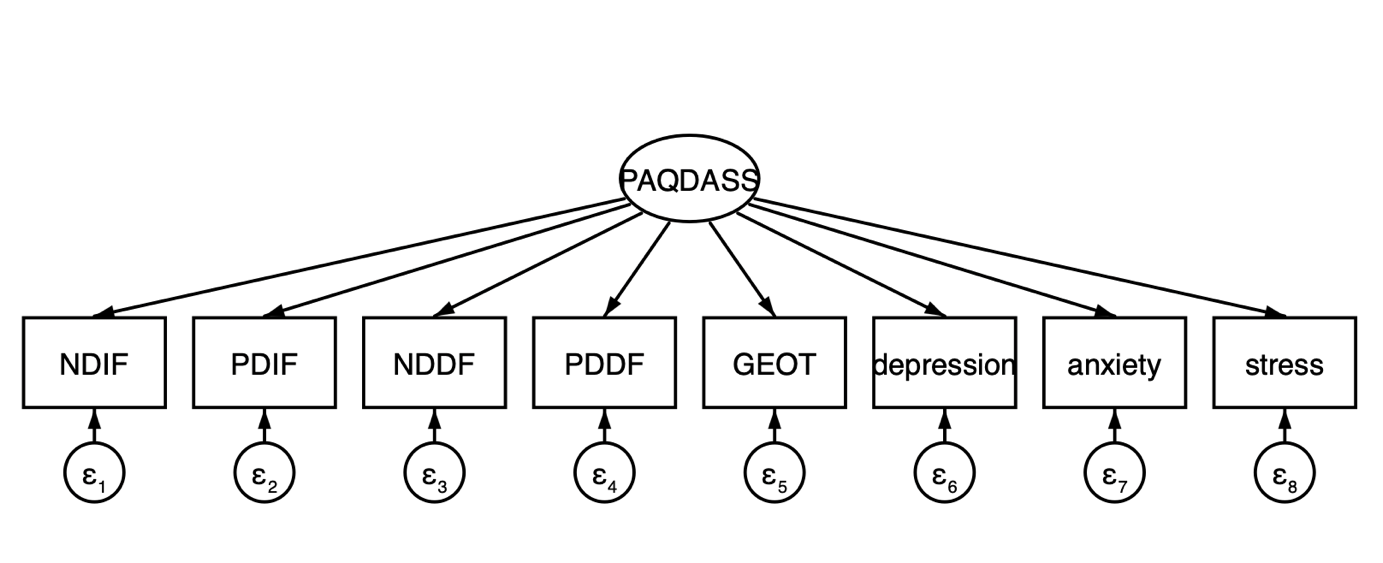


Model 3 :


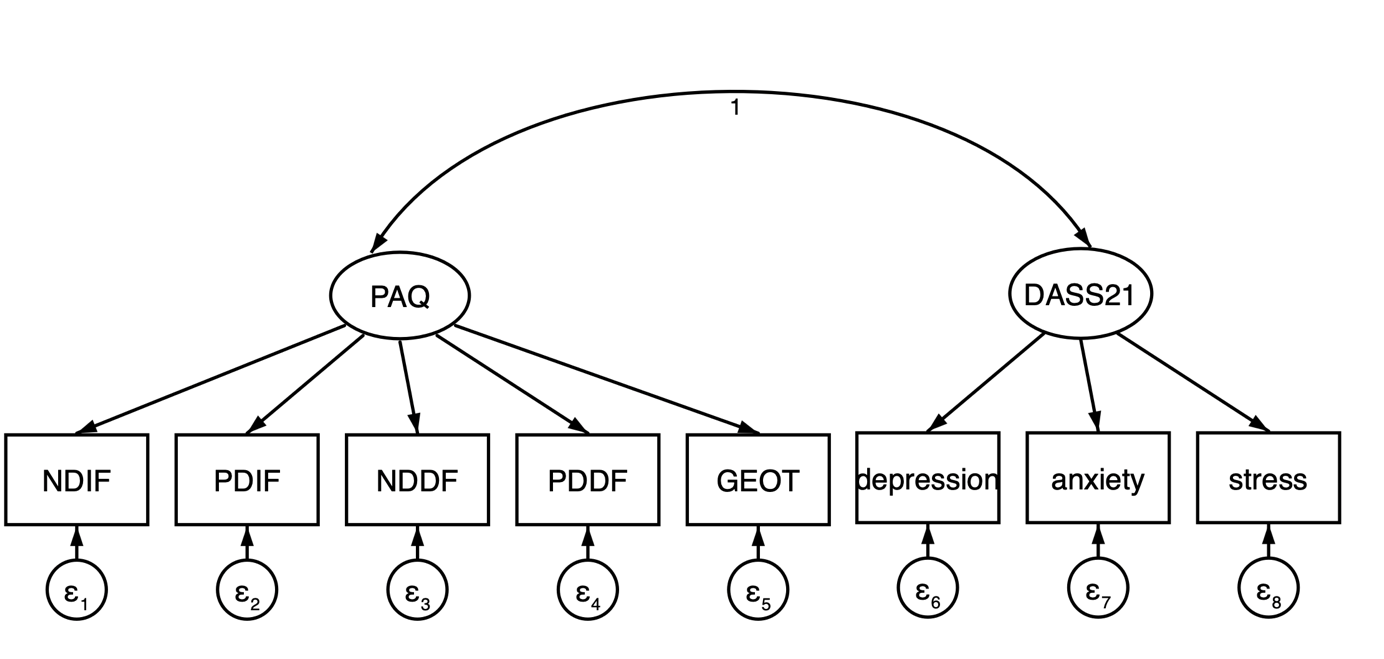

Supplement: Supplementary file 1 [file Supplementary_file_1.docx]
